# Response to correspondence regarding “The value of micro-ultrasound for prostate cancer screening: a retrospective real-world feasibility study”

**DOI:** 10.1038/s41391-026-01112-9

**Published:** 2026-03-26

**Authors:** Johann J. Wendler, Jonas Bechstein, Hannes Cash

**Affiliations:** 1 Clinic of Urology, Urooncology and Robotic and Focal Therapy, Medical Faculty of University Magdeburg and University Hospital Magdeburg A. ö. R. (University Medicine Magdeburg, UMM), Magdeburg, Germany; 2 Urologicum Magdeburg, Magdeburg, Germany; 3 PROURO Berlin, Berlin, Germany

**Keywords:** Outcomes research, Cancer screening

**To the Editor**,

We thank Wang et al. for their thoughtful engagement with our recent study on micro-ultrasound (MUS) in prostate cancer (PCa) screening [1] and welcome the opportunity to clarify several methodological aspects.

Wang et al. [2] raise concerns regarding potential verification bias due to the relatively low proportion of participants undergoing biopsy as the pathological gold standard, emphasizing the resulting limitations for estimation of negative predictive value (NPV) in real-world PCa screening cohorts. As in most contemporary screening trials, including the Göteborg screening study, the diagnostic pathway was guided by ethical and clinical considerations rather than systematic verification of all participants [3]. Consequently, biopsy was not performed universally, reflecting the accepted structure of pragmatic screening pathways rather than a methodological limitation. We see no need to further discuss the accepted role of a low prostate-specific antigen threshold below 2 ng/ml as a clinical ground truth for very low cancer risk.

Negative MUS or prostate-specific antigen (PSA) density (PSAD)-modified negative PRI-MUS (prostate risk identification using MUS) findings were not intended as definitive exclusion criteria for further diagnostic work-up but rather as pragmatic tools aimed at reducing unnecessary magnetic resonance imaging (MRI) and biopsy procedures while preserving detection of clinically significant PCa (csPCa). Accordingly, the reported NPV reflects outcomes within the implemented clinical pathway and represents decision safety rather than absolute pathological exclusion across the entire screening population.

Longitudinal follow-up represents an important next step. Prospective extensions, including structured PSA surveillance and interval imaging (MUS and/or MRI), will be essential to assess long-term safety. Similar verification constraints are inherent to MRI-based PCa screening studies, where complete pathological confirmation of all screen-negative participants is rarely feasible, ethically justified, or reflective of real-world clinical practice [4–6].

Wang et al. further argue that physical limitations of MUS might increase the risk of depth-related false negatives, particularly in larger glands or anterior regions. The physical trade-off between resolution and penetration depth in high-frequency ultrasound is well recognized. However, anterior visualization is influenced not solely by prostate volume but also by anatomical orientation, scanning technique, and acoustic shadowing, especially due to calcifications. In our cohort, the majority of csPCa detected by MRI and/or biopsy occurred in prostates with volumes below 60 mL, with median volumes of 32 mL [interquartile range (IQR) 25–41.5 mL; MUS] and 43 mL [IQR 32–67 mL; MRI], reflecting typical screening populations rather than extreme enlargement. Furthermore, trials focusing on cancer detection have demonstrated robust anterior lesion detection, and the prospective OPTIMUM trial showed an overall detection advantage compared with MRI [7, 8]. Therefore, aside from cases with extreme benign prostatic hyperplasia or extensive prostatic calcifications, we do not share the view that anterior imaging represents a general limitation of MUS.

Stratified analyzes by prostate volume remain limited for MUS but are similarly scarce for MRI, while the established clinical role of PSAD requires no further elaboration.

We envision a clinical workflow in which MUS or PSAD-modified MUS serves as a first-line triage tool, with MRI deployed selectively in cases of poor acoustic access or large gland volumes rather than applying strict exclusion thresholds, thereby supporting resource-efficient and patient-centered screening paradigms.

Concerns were also raised regarding patient-level agreement between MUS and MRI. Wang et al. emphasize the importance of spatial precision in lesion targeting; however, patient-level agreement was intentionally chosen to reflect real-world clinical decision pathways. In opportunistic screening settings, the clinically relevant endpoint is detection of csPCa rather than exact spatial concordance between imaging modalities. Lesion-level discordance is biologically plausible given fundamental differences in imaging physics and tumor phenotype sensitivity, and should not be interpreted as evidence of inferior diagnostic performance. Published data indicate lesion concordance rates between MUS and MRI of approximately 84–96% in men at risk, although prospective evaluation in screening populations remains warranted.

Importantly, the PROSTAMUS study was intentionally designed as a pragmatic real-world feasibility assessment rather than a diagnostic accuracy or modality equivalence trial. The primary objective was the evaluation of clinical decision pathways within an opportunistic screening context where MRI is not universally implemented as first-line imaging. Within such frameworks, NPV reflects clinical decision safety rather than absolute disease exclusion, as partial verification is inherent to ethical screening strategies.

Moreover, discordance between MUS and MRI is biologically and physically plausible due to fundamentally different imaging principles and tumor phenotype sensitivities. MRI itself is not a pathological gold standard, and imaging-driven verification strategies may introduce incorporation bias through targeted biopsy pathways. Consequently, detection of csPCa—rather than spatial identity of imaging targets—represents the clinically meaningful endpoint in a screening pathway.

We appreciate the insightful discussion by Wang et al., as considerations regarding verification frameworks, acoustic physics, and spatial interpretation will contribute to the continued refinement of imaging-based screening strategies. Within this context, our findings support MUS as a promising and scalable triage modality capable of refining risk stratification and reducing unnecessary diagnostic work-up.

Future prospective studies will be essential to further define and consolidate the clinical role of MUS in prostate cancer screening pathways.
